# The Ecuadorian Spanish version of the Juvenile Arthritis Multidimensional Assessment Report (JAMAR)

**DOI:** 10.1007/s00296-018-3947-y

**Published:** 2018-04-07

**Authors:** Cristina Herrera Mora, Stella Maris Garay, Alessandro Consolaro, Francesca Bovis, Nicolino Ruperto, for the Paediatric Rheumatology International Trials Organisation (PRINTO)

**Affiliations:** 1 Hospital de Niños Roberto Gilbert Elizalde, Reumatología, Cdla. Atarazana, Av. Roberto Gilbert y Nicasio Safadi, 090514 Guayaquil, Ecuador; 2 Hospital Sor María Ludovica, Servicio de Reumatologia, La Plata, Argentina; 3 Clinica Pediatrica e Reumatologia, Paediatric Rheumatology International Trials Organisation (PRINTO), Istituto Giannina Gaslini, Via Gaslini 5, 16147 Genoa, Italy; 4 Dipartimento di Pediatria, Università di Genova, Genoa, Italy

**Keywords:** Juvenile idiopathic arthritis, Disease status, Functional ability, Health-related quality of life, JAMAR

## Abstract

The Juvenile Arthritis Multidimensional Assessment Report (JAMAR) is a new parent/patient reported outcome measure that enables a thorough assessment of the disease status in children with juvenile idiopathic arthritis (JIA). We report the results of the cross-cultural adaptation and validation of the parent and patient versions of the JAMAR in the Ecuadorian Spanish language. The reading comprehension of the questionnaire was tested in 10 JIA parents and patients. Each participating centre was asked to collect demographic, clinical data and the JAMAR in 100 consecutive JIA patients or all consecutive patients seen in a 6-month period and to administer the JAMAR to 100 healthy children and their parents. The statistical validation phase explored descriptive statistics and the psychometric issues of the JAMAR: the 3 Likert assumptions, floor/ceiling effects, internal consistency, Cronbach’s alpha, interscale correlations, test–retest reliability, and construct validity (convergent and discriminant validity). A total of 23 JIA patients (17.4% systemic, 17.4% RF negative poly-arthritis, 17.4% RF positive poly-arthritis, and 47.8% other categories) and 23 healthy children were enrolled in the paediatric centre of Guayaquil. The JAMAR components discriminated well healthy subjects from JIA patients. Notably, there is no significant difference between the healthy subjects and their affected peers in the school-related problems variable. All JAMAR components revealed good psychometric performances. In conclusion, the Ecuadorian Spanish version of the JAMAR is a valid tool for the assessment of children with JIA and is suitable for use both in routine clinical practice and clinical research.

## Introduction

The aim of the present study was to cross-culturally adapt and validate the Ecuadorian Spanish parent, child/adult version of the Juvenile Arthritis Multidimensional Assessment Report (JAMAR) [1] in patients with juvenile idiopathic arthritis (JIA). The JAMAR assesses the most relevant parent/patient reported outcomes in JIA, including overall well-being, functional status, health-related quality of life (HRQoL), pain, morning stiffness, disease activity/status/course, articular and extra-articular involvement, drug-related side effects/compliance, and satisfaction with illness outcome.

This project was part of a larger multinational study conducted by the Paediatric Rheumatology International Trials Organisation (PRINTO) [2] aimed to evaluate the epidemiology, outcome and treatment of childhood arthritis (EPOCA) in different geographic areas [3].

We report herein the results of the cross-cultural adaptation and validation of the parent and patient versions of the JAMAR in the Ecuadorian Spanish language.

## Materials and methods

The methodology employed has been described in detail in the introductory paper of the supplement [4]. In brief, it was a cross-sectional study of JIA children, classified according to the ILAR criteria [5, 6] and enrolled from April 2016 to January 2017. Children were recruited after Ethics Committee approval and consent from at least one parent.

### The JAMAR

The JAMAR [1] includes the following 15 sections:

1. Assessment of physical function (PF) using 15-items in which the ability of the child to perform each task is scored as follows: 0 = without difficulty, 1 = with some difficulty, 2 = with much difficulty, and 3 = unable to do and not applicable if it was not possible to answer the question or the patient was unable to perform the task due to their young age or to reasons other than JIA. The total PF score ranges from 0 to 45 and has 3 components: PF-lower limbs (PF-LL), PF-hand and wrist (PF-HW), and PF-upper segment (PF-US) each scoring from 0 to 15 [7]. Higher scores indicate higher degree of disability [8–10].
2. Rating of the intensity of the patient’s pain on a 21-numbered circle visual analogue scale (VAS) [11].
3. Assessment of the presence of joint pain or swelling (present/absent for each joint).
4. Assessment of morning stiffness (present/absent).
5. Assessment of extra-articular symptoms (fever and rash) (present/absent).
6. Rating of the level of disease activity on a 21-circle VAS.
7. Rating of disease status at the time of the visit (categorical scale).
8. Rating of disease course from previous visit (categorical scale).
9. Checklist of the medications the patient is taking (list of choices).
10. Checklist of side effects of medications.
11. Report of difficulties with medication administration (list of items).
12. Report of school/university/work problems caused by the disease (list of items).
13. Assessment of HRQoL, through the Physical Health (PhH), and Psychosocial Health (PsH) sub-scales (5 items each), and a total score. The four-point Likert response, referring to the prior month, is ‘never’ (score = 0), ‘sometimes’ (score = 1), ‘most of the time’ (score = 2), and ‘all the time’ (score = 3). A ‘not assessable’ column was included in the parent version of the questionnaire to designate questions that cannot be answered because of developmental immaturity. The total HRQoL score ranges from 0 to 30, with higher scores indicating worse HRQoL. A separate score for PhH and PsH (range 0–15) can be calculated [12–14].
14. Rating of the patient’s overall well-being on a 21-numbered circle VAS.
15. A question about satisfaction with the outcome of the illness (Yes/No) [15].

The JAMAR is available in three versions, one for parent proxy-report (child’s age 2–18), one for child self-report, with the suggested age range of 7–18 years, and one for adults.

### Cross-cultural adaptation and validation

The process of cross-cultural adaptation was conducted according to international guidelines with 2–3 forward and backward translations. In those countries for which the translation of JAMAR had been already cross-cultural adapted in a similar language (i.e., Spanish in South American countries), only the probe technique was performed. Reading comprehension and understanding of the translated questionnaires were tested in a probe sample of 10 JIA parents and 10 patients.

Each participating centre was asked to collect demographic, clinical data, and the JAMAR in 100 consecutive JIA patients or all consecutive patients seen in a 6-month period and to administer the JAMAR to 100 healthy children and their parents.

The statistical validation phase explored the descriptive statistics and the psychometric issues [16]. In particular, we evaluated the following validity components: the first Likert assumption [mean and standard deviation (SD) equivalence]; the second Likert assumption or equal item-scale correlations (Pearson r: all items within a scale should contribute equally to the total score); third Likert assumption (item internal consistency or linearity for which each item of a scale should be linearly related to the total score that is 90% of the items should have Pearson *r* ≥ 0.4); floor/ceiling effects (frequency of items at lower and higher extremes of the scales, respectively); internal consistency, measured by the Cronbach’s alpha, interscale correlation (the correlation between two scales should be lower than their reliability coefficients, as measured by Cronbach’s alpha); test–retest reliability or intra-class correlation coefficient (reproducibility of the JAMAR repeated after 1 or 2 weeks); and construct validity in its two components: the convergent or external validity which examines the correlation of the JAMAR sub-scales with the 6 JIA core-set variables, with the addition of the parent assessment of disease activity and pain by the Spearman’s correlation coefficients (r) [17] and the discriminant validity, which assesses whether the JAMAR discriminates between the different JIA categories and healthy children [18]. Quantitative data were reported as medians with first and third quartiles and categorical data as absolute frequencies and percentages.

The complete Ecuadorian.

Spanish parent and patient versions of the JAMAR are available upon request to PRINTO.

## Results

### Cross-cultural adaptation

The Ecuadorian Spanish JAMAR was cross-culturally adapted from the Argentinian Spanish version.

All 123 lines of the parent version of the JAMAR were understood by at least 80% of the 10 parents tested (median = 100%; range: 90–100%). All the 120 lines of the patient version of the JAMAR were understood by at least 80% of the children (median = 100%; range: 90–100%). Both versions of the JAMAR were unmodified after the probe technique.

### Demographic and clinical characteristics of the subjects

A total of 23 JIA patients and 23 healthy children (total of 46 subjects) were enrolled at the paediatric rheumatology centre of Guayaquil.

In the 23 JIA subjects, the JIA categories were 17.4% with systemic arthritis, 4.3% with oligoarthritis, 17.4% with RF negative poly-arthritis, 17.4% with RF positive poly-arthritis, 8.7% with enthesitis-related arthritis, and 34.8% with undifferentiated arthritis. Notably, none of the enrolled JIA patients is affected with psoriatic arthritis (Table 1).

**Table 1:** Descriptive statistics (medians, first and third quartiles or absolute frequencies and %) for the 23 JIA patients

	Systemic	Oligoarthritis	RF- poly-arthritis	RF + poly-arthritis	Enthesitis-related arthritis	Undifferentiated arthritis	All JIA patients	Healthy
	*N* = 4	*N* = 1	*N* = 4	*N* = 4	*N* = 2	*N* = 8	*N* = 23	*N* = 23
Female	3 (75%)	1 (100%)	2 (50%)	4 (100%)	0 (0%)	6 (75%)	16 (69.6%)	10 (43.5%)
Age at visit	11.5 (8.6–14)	9.2 (9.2–9.2)	12 (8.8–14.6)	13 (11.3–14.5)	15.5 (14.6–16.4)	14.3 (11.7–15.6)	14.3 (10.9–14.7)	11.6 (9.9–14.3)
Age at onset	4.9 (4.5–10.5)	8.5 (8.5–8.5)	3.1 (2.2–8.8)	10.5 (9.7–12.1)	13.5 (11.9–15)	11 (9.9–12.5)	10.6 (5–12.7)	
Disease duration	4.2 (1-6.6)	0.7 (0.7–0.7)	5.7 (3.3–9.2)	1.5 (1–3)	2 (1.3–2.7)	1.5 (0.8–4.7)	1.8 (1–5.2)	
ESR	18 (11.5–22.5)	28 (28–28)	26 (20–36)	15 (10–26)	7 (7–7)	22 (9–29)	22 (9–28)	
MD VAS (0–10 cm)	2.5 (1-3.5)	1.5 (1.5–1.5)	0 (0-1.5)	1.5 (0.8–2.3)	0 (0–0)	0 (0-1.8)	0 (0–2)	
No. swollen joints	0.5 (0–2)	0 (0–0)	0 (0–1)	0 (0-0.5)	0 (0–0)	0 (0–1)	0 (0–1)	
No. joints with pain	0.5 (0-1.5)	1 (1–1)	0 (0–1)	1 (0.5–1)	0 (0–0)	0.5 (0–2)	0 (0–1)	
No. joints with LOM	0 (0-4.5)	0 (0–0)	2.5 (0-6.5)	1 (0.5–1.5)	0 (0–0)	0.5 (0-2.5)	0 (0–2)	
No. active joints	0.5 (0–2)	0 (0–0)	0 (0–1)	0.5 (0–1)	0 (0–0)	1 (0–2)	0 (0–1)	
Active systemic features	3 (75%)	0 (0%)	0 (0%)	0 (0%)	0 (0%)	0 (0%)	3 (13%)*	
ANA status	0 (0%)	0 (0%)	0 (0%)	0 (0%)	0 (0%)	0 (0%)	0 (0%)	
Uveitis	0 (0%)	0 (0%)	0 (0%)	0 (0%)	0 (0%)	0 (0%)	0 (0%)	
PF Total Score	0.5 (0–8)	1 (1–1)	5.5 (2–7.5)	0 (0–0)	0 (0–0)	0 (0–12.5)	0 (0–7)	0 (0–1)*
Pain VAS	2.3 (0.5–4.8)	2 (2–2)	3.5 (1.3–7)	2.5 (0.5–4.5)	0.8 (0–1.5)	0.3 (0–1.3)	1 (0–4)	0 (0–0)*
Disease Activity VAS	1.8 (0-5.3)	2 (2–2)	2.3 (0.5–4)	1.8 (0.5–3.3)	0 (0–0)	0.3 (0–1.5)	0.5 (0–3.5)	
Well-being VAS	2 (0.3–6.3)	0 (0–0)	2.5 (1–4)	1.5 (0.5–3.3)	0 (0–0)	0 (0–2)	1 (0–3.5)	
HRQoL-PhH	3 (1.5–5.5)	2 (2–2)	4 (3–4.5)	2.5 (1–4.5)	0 (0–0)	0.5 (0–5)	2 (0–5)	0 (0–1)*
HRQoL-PsH	1 (0.5-3)	3 (3–3)	2.5 (1–3.5)	0 (0–1.5)	0 (0–0)	0 (0–3)	0 (0–3)	0 (0–2)*
HRQoL Total Score	4 (2-8.5)	5 (5–5)	7 (4–8)	2.5 (1–6)	0 (0–0)	0.5 (0–8)	3 (0–8)	0 (0–3)*
Pain/swell in > 1 joint	2 (50%)	1 (100%)	3 (75%)	3 (75%)	0 (0%)	4 (50%)	13 (56.5%)	1 (4.3%)**
Morning stiffness > 15 min	0 (0%)	0 (0%)	0 (0%)	2 (50%)	0 (0%)	1 (12.5%)	3 (13%)	
Subjective remission	2 (50%)	1 (100%)	2 (50%)	2 (50%)	0 (0%)	2 (25%)	9 (39.1%)	
In treatment	4 (100%)	1 (100%)	4 (100%)	4 (100%)	2 (100%)	8 (100%)	23 (100%)	
Reporting side effects	2 (50%)	1 (100%)	2 (50%)	0 (0%)	2 (100%)	2 (25%)	9 (39.1%)	
Taking medication regularly	4 (100%)	1 (100%)	3 (75%)	4 (100%)	2 (100%)	8 (100%)	22 (95.7%)	
With problems attending school	1 (33.3%)	0 (0%)		0 (0%)	0 (0%)	0 (0%)	1 (7.1%)	0 (0%)
Satisfied with disease outcome	3 (75%)	1 (100%)	2 (50%)	4 (100%)	2 (100%)	6 (75%)	18 (78.3%)	

Data related to the JAMAR refers to the 23 JIA patients and to the 23 healthy subjects for whom the questionnaire has been completed by the parents. *p* values refers to the comparison of the different JIA categories or to JIA versus healthy. *p < 0.05 **p < 0.001

*JAMAR* Juvenile Arthritis Multidimensional Assessment Report, *ESR* erythrocyte sedimentation rate, *MD* medical doctor, *VAS* visual analogue scale (score 0–10; 0 = no activity, 10 = maximum activity), *LOM* limitation of motion, *ANA* anti-nuclear antibodies, *PF* physical function (total score ranges from 0 to 45), *HRQoL* health-related quality of life (total score ranges from 0 to 30), *PhH* physical health (total score ranges from 0 to 15), *PsH* psychosocial health (total score ranges from 0 to 15)

All the 46 subjects had the parent version of the JAMAR completed by a parent (23 from parents of JIA patients and 23 from parents of healthy children). The JAMAR was completed by 41/46 (89.1%) mothers and 5/46 (10.9%) fathers. The child version of the JAMAR was completed by all the 46 children age 6 or older. In addition, patients younger than 7 years, capable to assess their personal condition and able to read and write, were asked to fill in the patient version of the questionnaire.

### Discriminant validity

The JAMAR results are presented in Table 1, including the scores [median (first–third quartiles)] obtained for the PF, the PhH, the PsH sub-scales, and total score of the HRQoL scales. The JAMAR components discriminated well between healthy subjects and JIA patients. Notably, there is no significant difference between the healthy subjects and their affected peers in the school-related problems.

In summary, the JAMAR revealed that JIA patients had a greater level of disability and pain, as well as a lower HRQoL than their healthy peers.

### Psychometric issues

The main psychometric properties of both parent and child versions of the JAMAR are reported in Table 2. The following “Results” section refers mainly to the parent’s version findings, unless otherwise specified.

**Table 2:** Main psychometric characteristics between the parent and child versions of the JAMAR

	Parent *N* = 23	Child *N* = 23
Missing values (first–third quartiles)	No missing values	No missing values
Response pattern	PF and HRQoL positively skewed	PF and HRQoL positively skewed
Floor effect, median		
PF	87.0%	91.3%
HRQoL-PhH	60.9%	69.6%
HRQoL-PsH	78.3%	73.9%
Pain VAS	30.4%	30.4%
Disease activity VAS	39.1%	34.8%
Well-being VAS	43.5%	39.1%
Ceiling effect, median		
PF	0.0%	0.0%
HRQoL-PhH	0.0%	0.0%
HRQoL-PsH	0.0%	0.0%
Pain VAS	0.0%	0.0%
Disease activity VAS	0.0%	0.0%
Well-being VAS	0.0%	0.0%
Items with equivalent item-scale correlation	67% for PF, 70% for HRQoL	87% for PF, 70% for HRQoL
Items with item-scale correlation ≥ 0.4	80% for PF, 70% for HRQoL	47% for PF,50% for HRQoL
Cronbach’s alpha		
PF-LL	0.98	0.96
PF-HW	0.79	0.54
PF-US	0.38	0.00
HRQoL-PhH	0.74	0.63
HRQoL-PsH	0.70	0.64
Items with item-scale correlation lower than the Cronbach alpha	87% for PF, 90% for HRQoL	80% for PF, 80% for HRQoL
Test–retest intra-class correlation		
PF total score	1.00	–
HRQoL- PhH	0.89	–
HRQoL- PsH	0.96	–
Spearman correlation with JIA core-set variables, median		
PF	0.4	0.4
HRQoL-PhH	0.4	0.4
HRQoL-PsH	0.3	0.2
Pain VAS	0.2	0.4
Disease activity VAS	0.4	0.2
Well-being VAS	0.4	0.4

*JAMAR* Juvenile Arthritis Multidimensional Assessment Report, *JIA* juvenile idiopathic arthritis, *VAS* visual analogue scale, *PF* physical function, *HRQoL* health-related quality of life, *PhH* physical health, *PsH* psychosocial health, *PF-LL* PF-lower limbs, *PF-HW* PF-hand and wrist, *PF-US* PF-upper segment

### Descriptive statistics (first Likert assumption)

There were no missing results for all JAMAR items, since data were collected through a web-based system that did not allow to skip answers and input of null values. The response pattern for both PF and HRQoL was positively skewed toward normal functional ability and normal HRQoL. A reduced number of response choices were used for all the different HRQoL items (with the exception of item 3), and for the all the PF items, except for PF items 1, 3, 4, and 5.

The mean and SD of the items within a scale were roughly equivalent for the PF and for the HRQoL items (data not shown). The median number of items marked as not applicable was 0% (0–0%) for the PF and for the HRQoL.

### Floor and ceiling effect

The median floor effect was 87% (78.3–91.3%) for the PF items, 60.9% (56.5–60.9%) for the HRQoL-PhH items, and 78.3% (69.6–78.3%) for the HRQoL-PsH items. The median ceiling effect was 0% (0–8.7%) for the PF items, 0% (0–0%) for the HRQoL-PhH items, and 0% (0–0%) for the HRQoL-PsH items. The median floor effect was 30.4% for the pain VAS, 39.1% for the disease activity VAS and 43.5% for the well-being VAS. The median ceiling effect was 0% for the pain VAS, 0% for the disease activity VAS, and 0% for the well-being VAS.

### Equal item-scale correlations (second Likert assumption)

Pearson item-scale correlations corrected for overlap were roughly equivalent for items within a scale for 67% of the PF items, with the exception of items 8, 9, 13, 14, and 15 and for 70% of the HRQoL items, with the exception of items 1, 2, and 6.

### Items internal consistency (third Likert assumption)

Pearson item-scale correlations were ≥ 0.4 for 80% of items of the PF (except for items 13, 14, and 15) and 70% of items of the HRQoL (except for items 1, 8, and 10).

### Cronbach’s alpha internal consistency

Cronbach’s alpha was 0.98 for PF-LL, 0.79 for PF-HW, and 0.38 for PF-US. Cronbach’s alpha was 0.74 for HRQoL-PhH and 0.70 for HRQoL-PsH.

### Interscale correlation

The Pearson correlation of each item of the PF and the HRQoL with all items included in the remaining scales of the questionnaires was lower than the Cronbach’s alpha, with the exception of the PF items 11 and 12 and of the HRQoL items 2 and 6 (data not shown).

### Test–retest reliability

Reliability was assessed in 10 JIA patients, by re-administering both versions (parent and child) of the JAMAR after a median of 7 days (range 7–7 days). The intra-class correlation coefficients (ICC) for the PF total score showed an almost perfect reproducibility (ICC = 1.0). The ICC for the HRQoL-PhH and for the HRQoL-PsH showed an almost perfect reproducibility (ICC = 0.89 and ICC = 0.96, respectively).

### Convergent validity

The Spearman correlation of the PF total score with the JIA core set of outcome variables ranged from 0.2 to 0.5 (median = 0.4). The PF total score best correlation was observed with the parent global assessment of well-being (*r* = 0.55, *p* = 0.006). For the HRQoL, the median correlation of the PhH with the JIA core set of outcome variables ranged from 0.4 to 0.7 (median = 0.4) whereas for the PsH ranged from 0.2 to 0.6 (median = 0.3). The HRQoL-PhH and the HRQoL-PsH showed the best correlation with the parent global assessment of well-being (*r* = 0.92, *p* < 0.001 and *r* = 0.76, *p* < 0.0001, respectively). The median correlations between the pain VAS, the well-being VAS, and the disease activity VAS and the physician-centered and laboratory measures were 0.2.

(0.1–0.3), 0.4 (0.3–0.4), 0.4 (0.2–0.5), respectively.

## Discussion

In this study, the Ecuadorian Spanish version of the JAMAR was cross-culturally adapted from the Argentinian Spanish version. According to the results of the validation analysis, the Ecuadorian Spanish parent and patient versions of the JAMAR possess satisfactory psychometric properties. The disease-specific components of the questionnaire discriminated well between patients with JIA and healthy controls. Notably, there was no significant difference between the healthy subjects and their affected peers in the school-related problem variable. This finding indicates that children with JIA adapt well to the consequences of JIA.

Psychometric performances were good for all domains of the JAMAR with some exceptions: 3 PF items (“Turn the head and look over the shoulders”, “Bend the head back and look at the ceiling” and “Bite into a sandwich or an apple”) and 3 HRQoL items (“Difficulty of taking care of him/herself”, “Having trouble getting along with other children” and “Appeared to be dissatisfied with his/her physical appearance or abilities”) showed a lower item’s internal consistency. However, the overall internal consistency was at least acceptable for all the domains, with the exception of the PF-US Cronbach’s alpha that was poor.

In the external validity evaluation, the Spearman’s correlations of the PF and HRQoL scores with JIA core-set parameters ranged from weak to moderate.

The results obtained for the parent version of the JAMAR are similar, although slightly poorer, to those obtained for the child version, which suggests that children are equally reliable proxy reporters of their disease and health status as their parents. However, we have to acknowledge that a limited sample of patients was recruited at the study Unit.

JAMAR is aimed to evaluate the side effects of medications and school attendance, which are other dimensions of daily life that were not previously considered by other HRQoL tools. This may provide useful information for intervention and follow-up in health care. In conclusion, the Ecuadorian Spanish version of the JAMAR was found to have satisfactory psychometric properties and it is, thus, a reliable and valid tool for the multidimensional assessment of children with JIA.

## References

[1] Filocamo G, Consolaro A, Schiappapietra B, Dalpra S, Lattanzi B, Magni-Manzoni S (2011). A new approach to clinical care of juvenile idiopathic arthritis: the Juvenile Arthritis Multidimensional Assessment Report. J Rheumatol.

[2] Ruperto N, Martini A (2011). Networking in paediatrics: the example of the paediatric rheumatology international trials organisation (PRINTO). Arch Dis Child.

[3] Consolaro A, Ruperto N, Filocamo G, Lanni S, Bracciolini G, Garrone M (2012). Seeking insights into the EPidemiology, treatment and Outcome of Childhood Arthritis through a multinational collaborative effort: introduction of the EPOCA study. Pediatr Rheumatol Online J.

[4] Bovis F, Consolaro A, Pistorio A, Garrone M, Scala S, Patrone E (2018). Cross-cultural adaptation and psychometric evaluation of the Juvenile Arthritis Multidimensional Assessment Report (JAMAR) in 54 languages across 52 countries: review of the general methodology. Rheumatol Int.

[5] Petty RE, Southwood TR, Baum J, Bhettay E, Glass DN, Manners P (1998). Revision of the proposed classification criteria for juvenile idiopathic arthritis: Durban, 1997. J Rheumatol.

[6] Petty RE, Southwood TR, Manners P, Baum J, Glass DN, Goldenberg J (2004). International league of associations for rheumatology classification of juvenile idiopathic arthritis: second revision, Edmonton, 2001. J Rheumatol.

[7] Filocamo G, Sztajnbok F, Cespedes-Cruz A, Magni-Manzoni S, Pistorio A, Viola S (2007). Development and validation of a new short and simple measure of physical function for juvenile idiopathic arthritis. Arthritis Rheum.

[8] Lovell DJ, Howe S, Shear E, Hartner S, McGirr G, Schulte M (1989). Development of a disability measurement tool for juvenile rheumatoid arthritis. The juvenile arthritis functional assessment scale. Arthritis Rheum.

[9] Howe S, Levinson J, Shear E, Hartner S, McGirr G, Schulte M (1991). Development of a disability measurement tool for juvenile rheumatoid arthritis. The juvenile arthritis functional assessment report for children and their parents. Arthritis Rheum.

[10] Singh G, Athreya BH, Fries JF, Goldsmith DP (1994). Measurement of health status in children with juvenile rheumatoid arthritis. Arthritis Rheum.

[11] Filocamo G, Davi S, Pistorio A, Bertamino M, Ruperto N, Lattanzi B (2010). Evaluation of 21-numbered circle and 10-centimeter horizontal line visual analog scales for physician and parent subjective ratings in juvenile idiopathic arthritis. J Rheumatol.

[12] Duffy CM, Arsenault L, Duffy KN, Paquin JD, Strawczynski H (1997). The juvenile arthritis quality of life questionnaire–development of a new responsive index for juvenile rheumatoid arthritis and juvenile spondyloarthritides. J Rheumatol.

[13] Varni JW, Seid M, Knight TS, Burwinkle T, Brown J, Szer IS (2002). The PedsQL(TM) in pediatric rheumatology—reliability, validity, and responsiveness of the pediatric quality of life inventory(TM) generic core scales and rheumatology module. Arthritis Rheum.

[14] Landgraf JM, Abetz L, Ware JE (1996). The CHQ user’s manual,.

[15] Filocamo G, Consolaro A, Schiappapietra B, Ruperto N, Pistorio A, Solari N (2012). Parent and child acceptable symptom state in juvenile idiopathic arthritis. J Rheumatol.

[16] Nunnally JC (1978). Psychometric theory.

[17] Giannini EH, Ruperto N, Ravelli A, Lovell DJ, Felson DT, Martini A (1997). Preliminary definition of improvement in juvenile arthritis. Arthritis Rheum.

[18] Ware JE, Harris WJ, Gandek B, Rogers BW, Reese PR (1997). MAP-R for windows: multitrait/multi-item analysis program—revised user’s guide. Version 1.0 ed.

